# *Lactobacillus salivarius* WZ1 Inhibits the Inflammatory Injury of Mouse Jejunum Caused by Enterotoxigenic *Escherichia coli* K88 by Regulating the TLR4/NF-κB/MyD88 Inflammatory Pathway and Gut Microbiota

**DOI:** 10.3390/microorganisms11030657

**Published:** 2023-03-03

**Authors:** Zhen Wei, Ziqi He, Tongyao Wang, Xiaoxuan Wang, Tiancheng Wang, Miao Long

**Affiliations:** Key Laboratory of Livestock Infectious Diseases, Ministry of Education, College of Animal Science & Veterinary Medicine, Shenyang Agricultural University, Shenyang 110866, China

**Keywords:** *Lactobacillus salivarius*, ETEC K88, gut microbiota, mice, inflammatory injury, TLR4/NF-κB/MyD88

## Abstract

Replacing antibiotics with probiotics has become an important way to safely and effectively prevent and treat some gastrointestinal diseases. This study was conducted to investigate whether *Lactobacillus salivarius* WZ1 (L.S) could reduce the inflammatory injury to the mouse jejunum induced by *Escherichia coli* (ETEC) K88. Forty Kunming mice were randomly divided into four groups with 10 mice in each group. From day 1 to day 14, the control group and the *E. coli* group were administered with normal saline each day, while the L.S group and the L.S + *E. coli* group were gavaged with *Lactobacillus salivarius* WZ1 1 × 10^8^ CFU/mL each day. On the 15th day, the *E. coli* group and the L.S + *E. coli* group were intragastrically administered ETEC K88 1 × 10^9^ CFU/mL and sacrificed 24 h later. Our results show that pretreatment with *Lactobacillus salivarius* WZ1 can dramatically protect the jejunum morphological structure from the changes caused by ETEC K88 and relieve the morphological lesions of the jejunum, inhibiting changes in the mRNA expressions of *TNF-α*, *IL-1β* and *IL-6* and the protein expressions of TLR4, NF-κB and MyD88 in the intestinal tissue of mice caused by ETEC K88. Moreover, pretreatment with *Lactobacillus salivarius* WZ1 also increased the relative abundance of beneficial genera such as *Lactobacillus* and *Bifidobacterium* and decreased the abundance of harmful genera such as *Ralstonia* and *Helicobacter* in the gut. These results demonstrate that *Lactobacillus salivarius* WZ1 can inhibit the inflammatory damage caused by ETEC K88 in mouse jejunum by regulating the TLR4/NF-κB/MyD88 inflammatory pathway and gut microbiota.

## 1. Introduction

*Escherichia coli* is the most common bacterial infection causing diarrhea in animals [1,2]. It causes diarrhea, hemorrhagic colitis and dysentery in weak, malnourished and immunocompromised calves, especially those that have not acquired maternal antibodies through colostrum feeding [3]. Diarrhea *Escherichia coli* can be divided into six categories according to virulence: enterotoxigenic *Escherichia coli* (ETEC), Shiga toxin-producing *Escherichia coli* (STEC), enteropathogenic *Escherichia coli* (EPEC), enteroinvasive *Escherichia coli* (EIEC), enteroaggregative *Escherichia coli* (EAEC) and enterohemorrhagic *Escherichia coli* (EHEC) [4]. Among them, ETEC is the most serious, and its pathogenic types have different specific adhesins and enterotoxins. Fimbriae adhesins K88 (F4), K99 (F5), 987P (F6) and F41 (F7) play an important role in ETEC infection in newborn animals. These virulence factors can colonize bacteria in intestinal mucosa and cause intestinal osmotic imbalance, resulting in diarrhea [5,6,7]. Infection is characterized by acute watery diarrhea, rapid dehydration and collapse within hours, vomiting, stomach cramps, headache and, rarely, mild fever [8]. ETEC infection can increase the concentration of inflammatory factors in serum, the jejunum and the colon, causing the body to produce an inflammatory response, reducing the expression of tight junction proteins and mRNA in the intestinal epithelium of the jejunum and colon, and destroying the intestinal barrier [9]. Currently, antibiotics are commonly used in production to treat diarrhea caused by *Escherichia coli*, but these antibiotics affect not only the target pathogens, but also the beneficial microbes in the gut, leading to the growth of the gut microbiota associated with the disease [10]. Furthermore, in many cases, producers add antibiotics to feed to reduce the chance of infection, which is non-therapeutic, irresponsible and leads to a rapid increase in resistant bacteria [11]. Therefore, determining antibiotic alternatives to prevent or treat diarrhea is a current research hotspot.

Probiotics are “live microorganisms that, when ingested in adequate amounts, are beneficial to the health of the host” [12]. Probiotics can compete for receptors and adhere to cells, then colonize and remain viable in the gut, effectively compete with pre-existing pathogenic microorganisms through the acidification and production of antimicrobial compounds, affect the enzymatic activation of bacterial toxin receptor modification, and improve the body’s immune defense against pathogenic microorganisms [13,14]. Therefore, many studies on the use of probiotics to replace antibiotics in the prevention or treatment of infectious diarrhea caused by pathogenic bacteria in livestock are underway.

*Lactobacillus salivarius* belongs to the genus *Lactobacillus* and the family *Lactobacillaceae*. It is a Gram-positive bacterium and an important member of the symbiotic flora of humans and animals [15]. *Lactobacillus salivarius* is frequently isolated from animal gut, human milk and other sources. It has the ability to modulate gut microbiota, produce antimicrobial substances, stimulate protective immune responses, inhibit fecal enzyme activity and produce the short-chain fatty acids that allow the gut to undergo acidification [16]. Experiments have shown that *Lactobacillus salivarius* UCC118 can secrete bacteriocin Abp118, which exhibits antibacterial activity and can inhibit the growth of various pathogenic bacteria [17]; heat-inactivated *Lactobacillus salivarius* CECT 5713 can inhibit *Streptococcus mutans* [18]; and *Lactobacillus salivarius* LS01 supernatant has inhibitory effects on *Escherichia coli* and *Staphylococcus aureus* [19]. Studies have shown that *Lactobacillus salivarius* has good application in improving animal health, such as in combating intestinal inflammation through the production and activity of regulatory T cells, macrophages, natural killer cells and dendritic cells [20]; reducing pro-inflammatory cytokines in mouse splenocytes [21]; improving muscle strength and endurance after exercise [22]; and significantly reducing fasting blood glucose levels in diabetic mice [23]. Therefore, *Lactobacillus salivarius* has been incorporated into microecological preparations, and using this bacterium in actual production has good development prospects.

The findings from our unpublished research suggest that *Lactobacillus salivarius* WZ1, isolated and identified by our laboratory, was resistant to acid and bile salts and had a good inhibitory effect on pathogenic bacteria such as *Escherichia coli*, *Salmonella*, *Staphylococcus aureus* and *Clostridium perfringens*. However, it is not clear whether this strain can prevent the intestinal damage caused by ETEC K88 in vivo and exert its protective mechanisms.

The purpose of this study was to investigate the mechanism of *Lactobacillus salivarius* WZ1 and its effect on ETEC K88–induced jejunum inflammatory injury in mice and to explore whether it could affect intestinal immune function and gut microbiota, thereby inhibiting the intestinal injury caused by ETEC K88. This lays a theoretical foundation for the clinical application of *Lactobacillus salivary* WZ1 in preventing calf diarrhea.

## 2. Materials and Methods

### 2.1. Strains and Culture Growth Conditions

*Lactobacillus salivarius* WZ1 (NCBI accession number: ON005140) was isolated from healthy calves, and ETEC K88 standard strain (CVCC196) was purchased from the China Culture Collection Center and preserved by our laboratory. *Lactobacillus salivarius* WZ1 was cultured in MRS broth (Solearbio, Beijing, China), and ETEC K88 was cultured in NB broth (Solearbio, Beijing, China) at 37 °C, 130 rpm/min for 24 h.

### 2.2. Test Grouping

Specific-pathogen-free (SPF) grade Kunming mice (6-week-old, *n* = 40, body weight 30 ± 2 g) were purchased from Liaoning Changsheng Biotechnology Co., Ltd. (Shenyang, China). Before the experiment, the mice were given a one-week acclimation period where the temperature was kept at 23 ± 2 °C and a 12 h light–dark cycle was used. The experiments were permitted by the Shenyang Agricultural University under the Laboratory Animal Care Ethics Committee (People’s Republic of China Animal Ethics Regulations and Guidelines) for animal experiments (Permit No. 264SYXK2011-0001, September 2018). A total of 40 Kunming mice were randomly divided into 4 groups: the CON group (blank control group, *n* = 10); the L.S group (*Lactobacillus salivarius* WZ1 group, *n* = 10); the *E. coli* group (ETEC K88 group, *n* = 10); and the L.S + *E. coli* group (*Lactobacillus salivarius* WZ1 + ETEC K88 group, *n* = 10). Fourteen days before the start of the experiment, the CON group and the *E. coli* group were administered normal saline each day, while the L.S group and the L.S + *E. coli* group were intragastrically administered *Lactobacillus salivarius* WZ1 1 × 10^8^ CFU/mL each day [24]. On the 15th day, the *E. coli* group and the L.S + *E. coli* group were intragastrically administered ETEC K88 1 × 10^9^ CFU/mL [25], while the CON group and the L.S group were gavaged with normal saline. The mice were sacrificed after 24 h [26].

### 2.3. Histological Evaluation (HE) of Jejunum

We took 10 mL centrifuge tubes, added 7 mL of tissue fixative to each tube, and marked them for future use. In the absence of mechanical damage, mouse jejunum tissue samples of approximately 1–2 cm were selected and placed in the prepared tissue fixative for measurement of villus length and crypt depth, followed by HE staining and observation with a microscope (Savier, Wuhan, China).

### 2.4. Detection of Inflammatory Factors by RT-qPCR

The tissue samples were placed on ice, and 0.1 g tissue samples were weighed with sterilized scissors and tweezers. The samples were placed into a sterilized mortar with a small amount of liquid nitrogen and quickly ground into powder with a grinding pestle. The powder was removed with a sterilizing spoon and inserted into a 1.5 mL deenzymatic centrifuge tube, and 1 mL RNA lysate was added to make tissue homogenate. A total RNA extraction kit (Novizan, Nanjing, China) was used to extract RNA from the mouse jejunum tissues. An amount of 12,000 g was centrifuged at 4 °C for 5 min, after which the supernatant was discarded and let dry. We dissolved the precipitate with 20 μL of sterile enzyme-free deionized water and used an instrument to detect the concentration of extracted RNA. Residual genomic DNA from RNA templates was removed (the loading volume was 1 ng/extracted RNA concentration), and the RNA was reverse transcribed into cDNA using HiScript^®^ III RT SuperMix for qPCR (+gDNA wiper) (Novizan, Nanjing, China). The cDNA obtained from the reaction was used to detect the mRNA expression of *TNF-α*, *IL-1β* and *IL-6* in the tissue using ChamQ Universal SYBR qPCR Master Mix (Novizan, Nanjing, China).

*GAPDH* was used as the internal reference primer for mRNA. The experimental results were expressed using the 2^−△△Ct^ method to express the changes in mRNA expression in the treatment group relative to the control group. The primers used in this study are listed in Table 1. RT-qPCR reaction procedure: stage 1: 95 °C, 30 s; stage 2: 95 °C, 10 s; 60 °C, 30 s; 95 °C, 15 s (34 ×); and stage 3: 60 °C, 60 s; 95 °C, 15 s.

### 2.5. Western Blot

Mice jejunal tissue proteins were extracted using a tissue whole protein extraction kit (Solearbio, Beijing, China), the concentrations of the extracted total protein were determined using the Omni-Easy^TM^ ready-to-use BCA protein quantification kit (Yase, Shanghai, China), and the formula was obtained: sample protein concentration = (OD 562 nm–0.1371)/0.7618. The proteins were separated and transferred to PVDF membranes using a PAGE gel rapid prep kit (Yase, Shanghai, China), and blocked with 5% nonfat dry milk (Solearbio, Beijing, China) for 2 h at room temperature on a shaker. After the primary antibodies (TLR4, NF-κB, MyD88, Occludin, ZO-1, Claudin-1) were prepared at a dilution ratio of 1:1000, the PVDF membranes were placed in the primary antibody and incubated at 4 °C with a shaker for 2 h. They were then washed with 1 × TBST, put in the secondary antibody (HRP* Goat Anti-Mouse IgG (H + L) and HRP * Goat Anti-Rabbit IgG (H + L)), and incubated at room temperature for 1 h on a shaker. Finally, we used the Omni-Easy^TM^ basic chemiluminescence detection kit (Yase, Shanghai, China) and incubated the samples at room temperature for 3 to 5 min in the dark. We employed the Shanghai Qinxiang CLiNX gel imaging system to observe the results, used Gel Quant software to detect the gray value of the target protein and the internal reference protein, and calculated the relative expression of the protein according to the calculation formula.

### 2.6. Analysis of Gut Microbiota

We prepared several sterile cryopreservation tubes, put the mouse cecum contents into cryopreservation tubes under aseptic conditions, immediately placed them into liquid nitrogen, and then set them at −80 °C for later use. After extracting the total DNA from the sample, primers were designed according to the conserved regions and sequencing adapters were added to the ends of the primers, PCR amplification was performed, and the products were purified, quantified and homogenized to form a sequencing library. Qualified libraries were sequenced with an Illumina HiSeq 2500 sequencing system (Beijing, BioMark Biotechnology Co., Ltd., Beijing, China). The sequencing results were analyzed using OTU, a species distribution histogram, alpha diversity, beta diversity (PCA, PCoA), LEfSe and ANOVA. The raw sequencing data generated from this study were deposited in NCBI SRA1 under the BioProject accession number PRJNA835604 (https://www.ncbi.nlm.nih.gov/sra/PRJNA835604 (accessed on 6 May 2022)).

### 2.7. Data Statistics and Analysis

**Figure 1:**
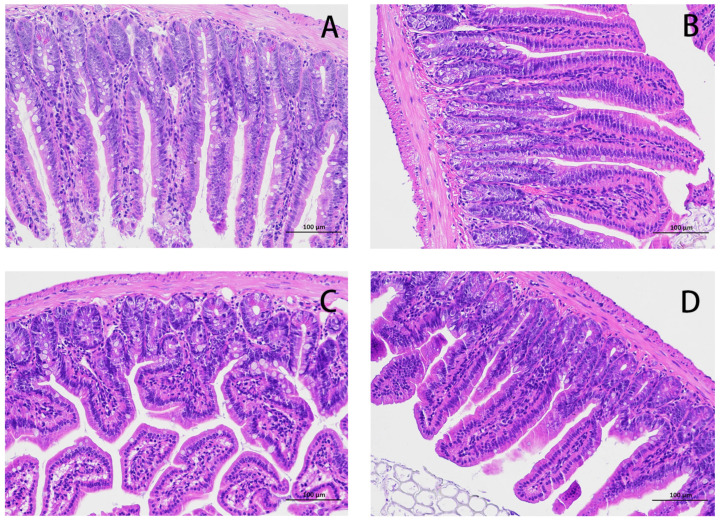
HE staining of mouse jejunum tissue (200×). Note: (**A**): CON group, (**B**): L.S group, (**C**): *E. coli* group, (**D**): L.S + *E. coli* group.

**Figure 2:**
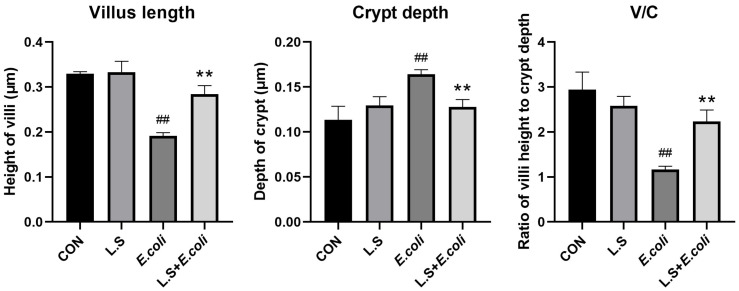
Villus length and crypt depth in mouse jejunum tissue and the ratio between them. Note: ## in the figure indicates a very significant difference compared with the CON group (*p* < 0.01); ** indicates a very significant difference compared with the *E. coli* group (*p* < 0.01).

**Figure 3:**
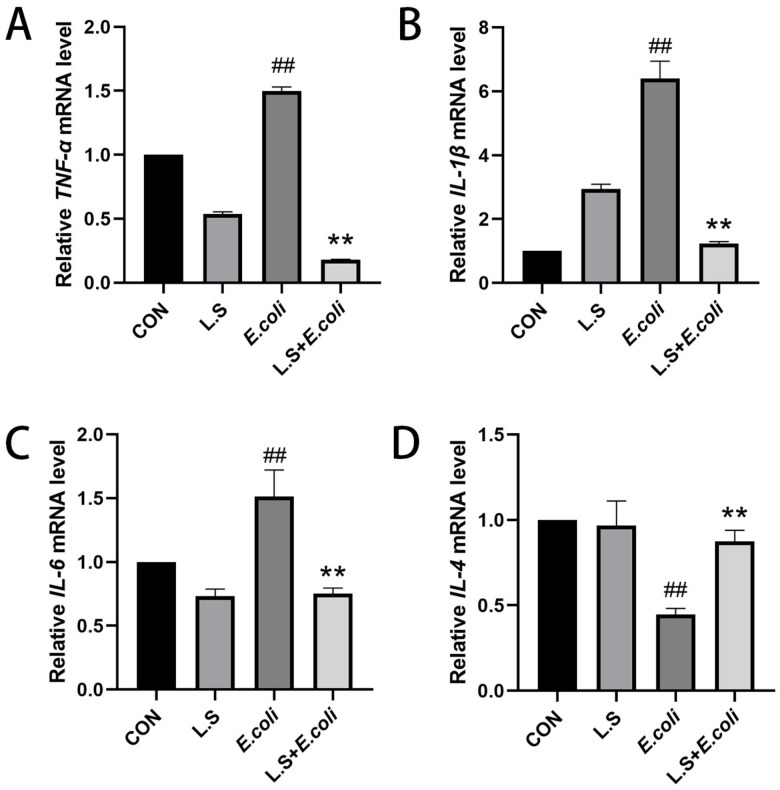
Relative expression of inflammatory factor mRNA in mouse jejunum tissue. Note: ## in the figure indicates a very significant difference compared with the CON group (*p* < 0.01); ** indicates a very significant difference compared with the *E. coli* group (*p* < 0.01). (**A**): Relative *TNF-α* mRNA expression, (**B**): Relative *IL-1β* mRNA expression, (**C**) Relative *IL-6* mRNA expression, (**D**) Relative *IL-4* mRNA expression.

**Figure 4:**
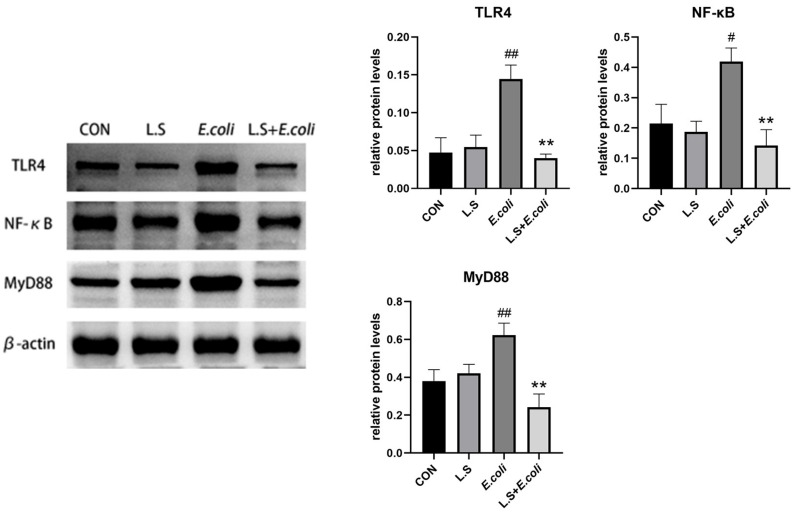
Expression of inflammation-related proteins in mouse jejunum. Note: # in the figure indicates a significant difference compared with the CON group (*p* < 0.05); ## indicates a very significant difference compared with the CON group (*p* < 0.01); ** indicates a difference with the *E. coli* group where the difference is extremely significant (*p* < 0.01).

**Figure 5:**
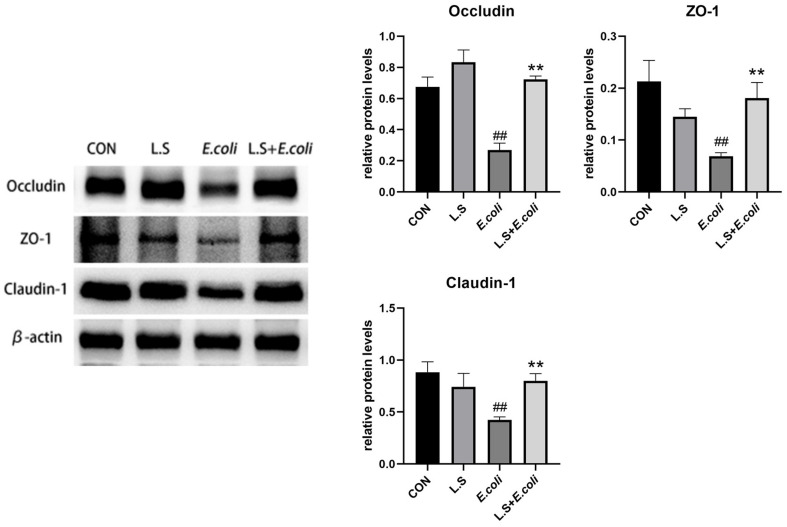
Expression of tight-junction-related proteins in the intestinal epithelium of the mouse jejunum. Note: ## in the figure indicates a very significant difference compared with the CON group (*p* < 0.01); ** indicates a very significant difference compared with the *E. coli* group (*p* < 0.01).

**Figure 6:**
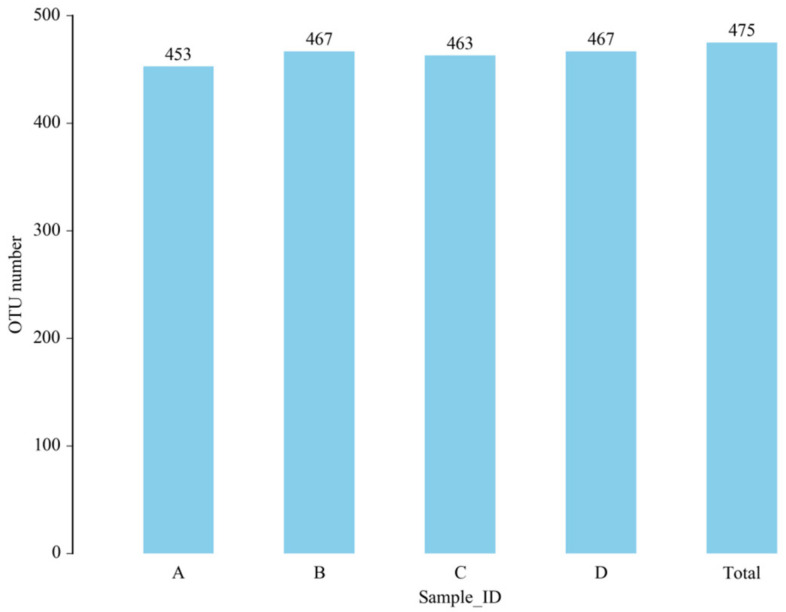
Number of OTUs. Note: A: CON group, B: L.S group, C: *E. coli* group, D: L.S + *E. coli* group.

**Figure 7:**
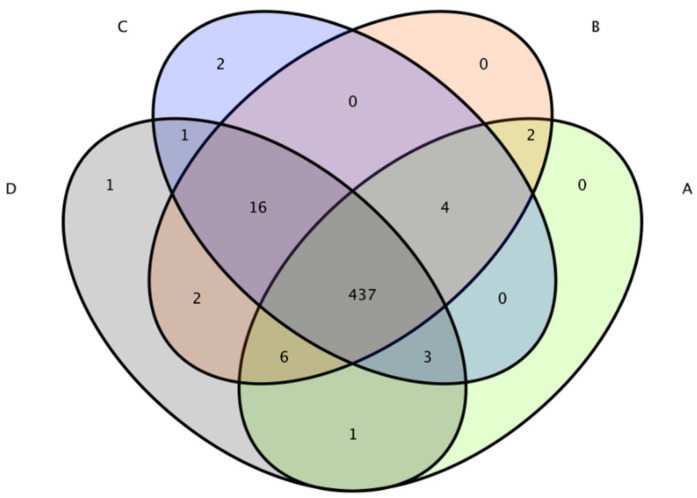
Venn diagram. Note: A: CON group, B: L.S group, C: *E. coli* group, D: L.S + *E. coli* group.

**Figure 8:**
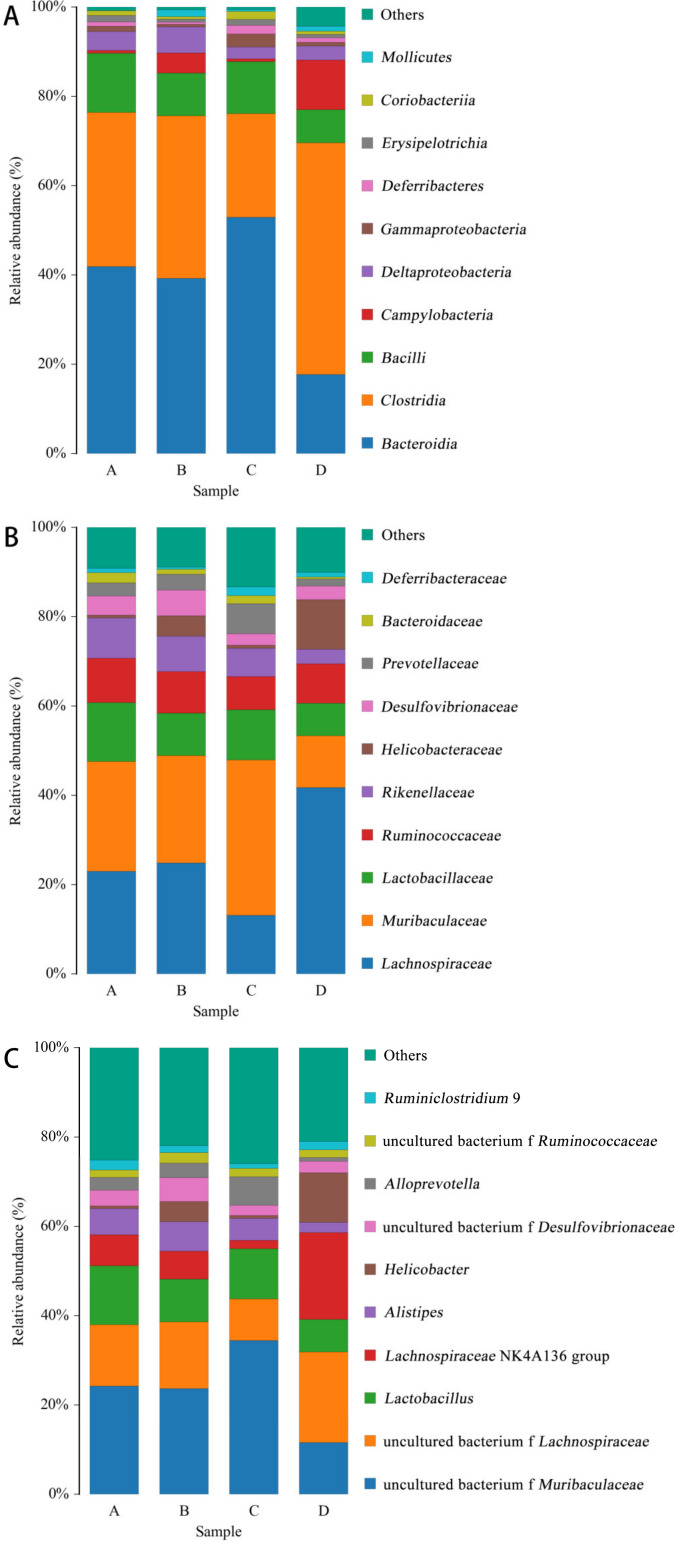
Species distribution histogram. Note: A: CON group, B: L.S group, C: *E.coli* group, D: L.S + *E. coli* group. (**A**): class level. (**B**): family level. (**C**): genus level.

**Figure 9:**
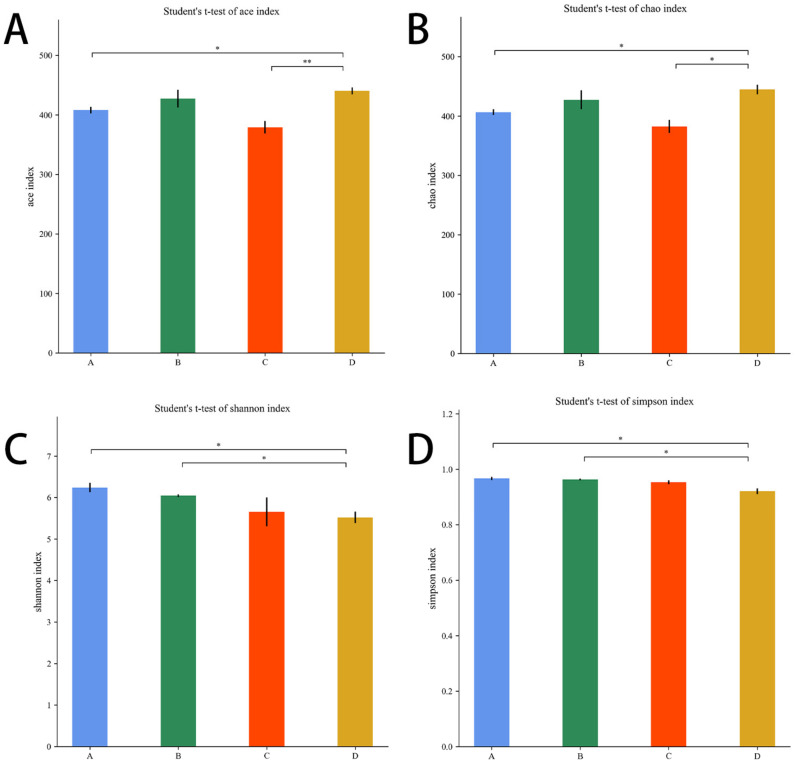
Alpha diversity index. Note: A: CON group, B: L.S group, C: *E. coli* group, D: L.S + *E. coli* group. * indicates significant difference (*p* < 0.05); ** indicates extremely significant difference (*p* < 0.01). (**A**): Ace index. (**B**): Chao1 index. (**C**): Shannon index .(**D**): Simpson index.

**Figure 10:**
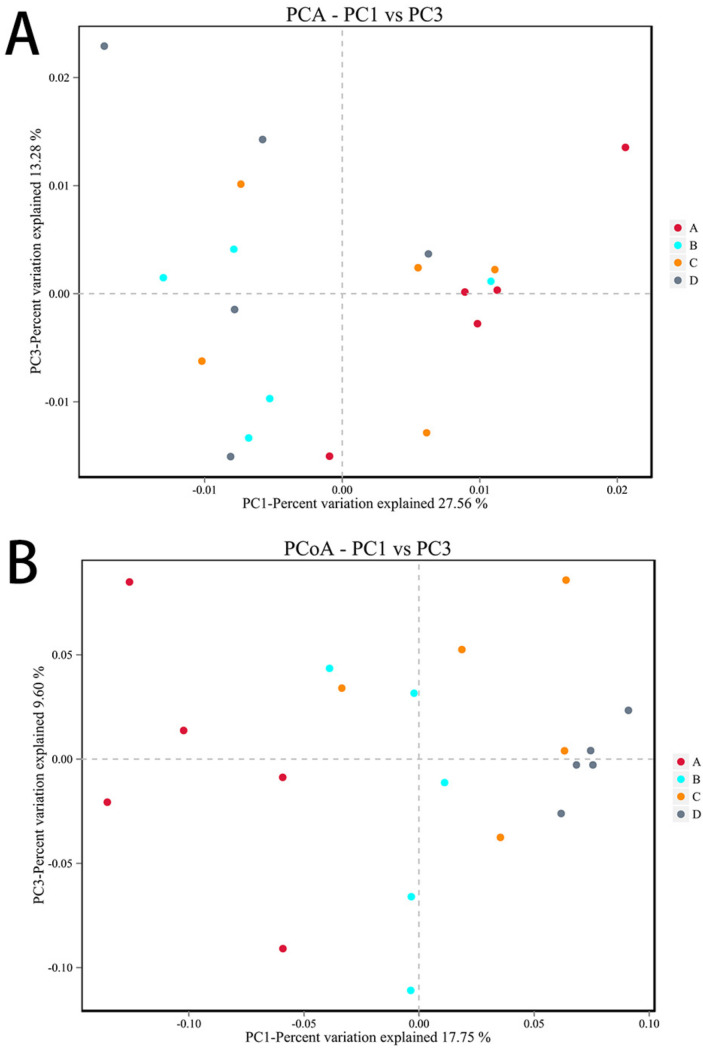
Beta diversity analysis. Note: Each data point represents a different sample (its color representing the group to which it belongs). The abscissa (ordinate) represents the first (second) principal component. The percentages shown are the contributions the component makes to the sample difference. A: CON group, B: L.S group, C: *E. coli* group, D: L.S + *E. coli* group. (**A**): PCA analyses (**B**): PCoA analyses.

**Figure 11:**
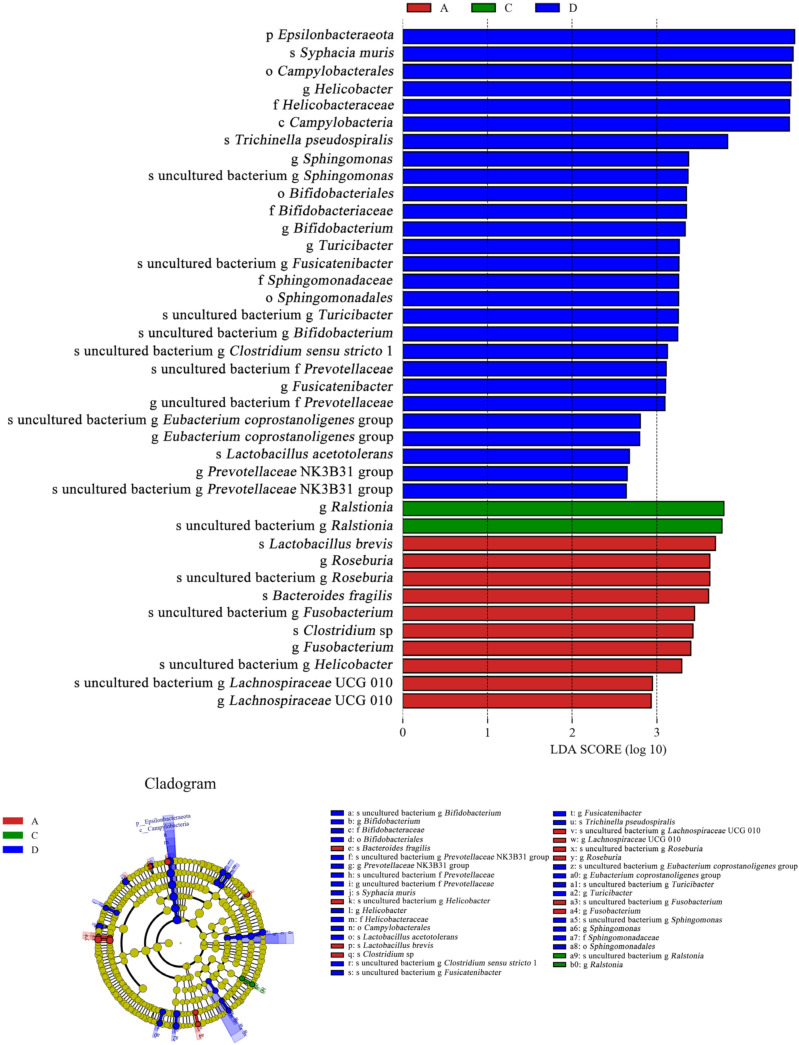
LEfSe analysis. Note: A: CON group, C: *E. coli* group, D: L.S + *E. coli* group.

**Figure 12:**
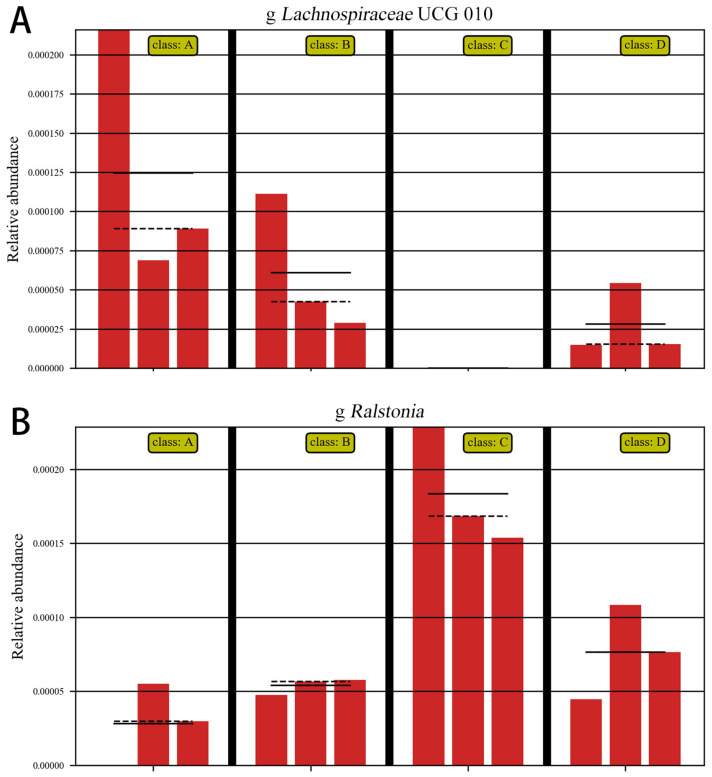
Abundance comparison chart. Note: A: CON group, B: L.S group, C: *E. coli* group, D: L.S + *E. coli* group. (**A**): the abundance of *Lachnospiraceae* (**B**): the abundance of *Ralstonia*.

**Figure 13:**
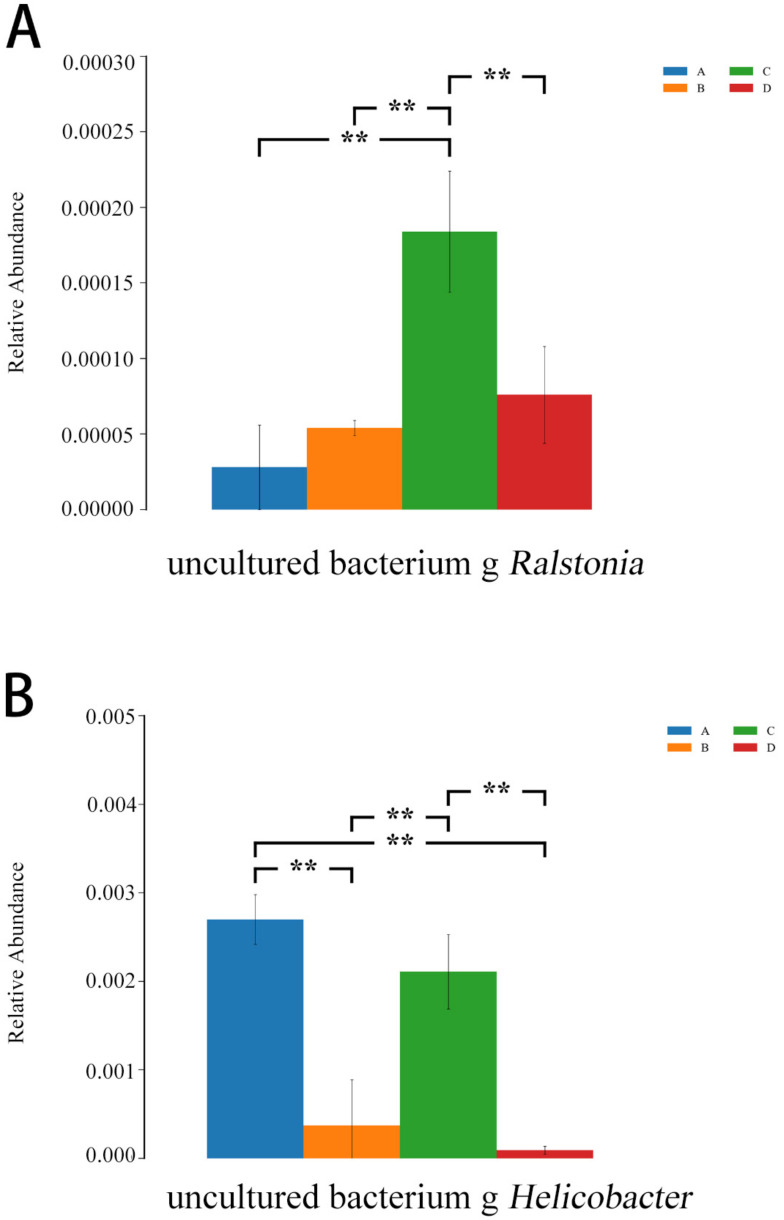
ANOVA analysis diagram. Note: A: CON group, B: L.S group, C: *E. coli* group, D: L.S + *E. coli* group. ** indicates extremely significant difference (*p* < 0.01). (**A**): the relative abundances of *Ralstonia* (**B**): the relative abundances of *Helicobacter*.

All experiments were independently repeated at least three times. All test data were preliminarily calculated and counted using Microsoft Excel, and then one-way variance calculation and analysis were carried out using SPSS data analysis software (Figures 2–5,9 and 13). The test values are expressed in the form of mean ± standard error (mean ± SE). The difference significance judgment took *p* < 0.05 as a significant difference and *p* < 0.01 as an extremely significant difference. The results of this test were calculated and plotted in Figures 1–5 using Graphpad Prism 8 and Office, and in Figures 6–13 using the microbial diversity analysis platform (BioMark Biotechnology Co., Ltd., Beijing, China).

## 3. Results

### 3.1. Pathological Changes in Jejunum

The HE results for the mouse jejunum are shown in Figure 1. The intestinal villi in the CON group were arranged in an orderly manner without rupture (Figure 1A). Compared with the CON group, the intestinal villi in the *E. coli* group were disordered and obviously broken (Figure 1C). Compared with the *E. coli* group, the intestinal villi in the L.S + *E. coli* group were arranged relatively neatly, and the fracture phenomenon was significantly reduced (Figure 1D). We measured the villus length and crypt depth of the small intestine and calculated the ratio of the two, with the results shown in Figure 2. The villus length in the *E. coli* group was shorter than that in the CON group (*p* < 0.01). The villus length in the L.S + *E. coli* group was longer than that in the *E. coli* group (*p* < 0.01). Compared with the CON group, the crypt depth of the *E. coli* group increased (*p* < 0.01), indicating that the stimulation of ETEC K88 led to a great increase in the crypt depth, resulting in a large number of immature cells with secretory functions, which caused diarrhea. The crypt depth of the L.S + *E. coli* group was significantly smaller than that of the *E. coli* group and had no significant difference from the CON group. The ratio between villus length and crypt depth (V/C) indicated the functional status of the intestine, and the decreased ratio indicated the decreased absorptive function and the increased secretory function of the intestine, thus causing diarrhea, while the increased ratio indicated the improved absorptive capacity of the intestine. The figure shows that the V/C of the *E. coli* group decreased significantly (*p* < 0.01), while the V/C of the L.S + *E. coli* group was significantly higher than that of the *E. coli* group (*p* < 0.01). In conclusion, *Lactobacillus salivarius* WZ1 can effectively inhibit the negative functional changes caused by ETEC K88 in the intestinal tract.

### 3.2. The mRNA Expression of Inflammatory Factors

As shown in Figure 3, the test results reveal that, compared with the CON group, the mRNA expressions of *TNF-α*, *IL-1β* and *IL-6* in the intestinal tissue of the *E. coli* group were increased (*p* < 0.01), and the mRNA expression of *IL-4* was decreased (*p* < 0.01), indicating that ETEC K88 could increase the secretion of the pro-inflammatory cytokines *TNF-α*, *IL-1β* and *IL-6* and decrease the secretion of anti-inflammatory factor IL-4 in the intestinal tract of the mice. Compared with the *E. coli* group, the mRNA expressions of *TNF-α*, *IL-1β* and *IL-6* in the intestinal tissue of the L.S + *E. coli* group were decreased (*p* < 0.01), and the mRNA expression of *IL-4* was increased (*p* < 0.01), indicating that feeding with *Lactobacillus salivarius* WZ1 can effectively prevent the inflammatory response to ETEC K88 in the intestinal tract of mice. The intestinal tract plays a significant protective role.

### 3.3. Inflammatory Pathway Protein Expression

As shown in Figure 4, compared with the control group, the protein expressions of TLR4, NF-κB and MyD88 in the jejunum in the *E. coli* group were significantly increased (*p* < 0.01), indicating that ETEC K88 activated the TLR4/NF-κB/MyD88 pathway, causing inflammatory damage to intestinal tissue. Compared with the *E. coli* group, the protein expressions of TLR4, NF-κB and MyD88 in the jejunum in the L.S + *E. coli* group were significantly decreased (*p* < 0.01). This result indicates that *Lactobacillus salivarius* WZ1 can effectively prevent the inflammatory damage caused by ETEC K88 to the intestinal tract.

### 3.4. Tight Junction Protein Expression

As shown in Figure 5, compared with the control group, ETEC K88 caused a significant decrease in the protein expressions of Occludin, ZO-1 and Claudin-1 in the jejunum (*p* < 0.01), and compared with the *E. coli* group, the expression of related proteins was significantly higher (*p* < 0.01). This result demonstrates that *Lactobacillus salivarius* WZ1 can promote intestinal epithelial tight junction protein production in intestinal cells, strengthen the intestinal mucosal barrier and effectively prevent the invasion of pathogenic bacteria.

### 3.5. Gut Microbiota Analysis

The microbial diversity of the mice cecum was based on the Illumina HiSeq sequencing platform, and paired-end cDNA libraries were constructed and sequenced (16 s, v3 + v4 b). First, we used Trimmatic v0.33 software to filter the raw reads obtained via sequencing; then, we used cutadapt 1.9.1 software to identify and remove the primer sequence, and obtain high-quality reads without primer sequences; afterwards, we used FLASH v1.2.7 software to splice the high-quality reads of each sample through overlay, and the resulting splicing sequence was the clean reads; finally, UCHIME v4.2 software was used to identify and remove the chimeric sequence to obtain the final effective data (effective reads).

In this study, 28 samples were sequenced and 2,239,382 pairs of reads were obtained. A total of 2,219,181 clean reads were generated after quality control and splicing. At least 79,040 clean reads were generated in each sample, with an average of 79,256 clean reads.

#### 3.5.1. OTU Analysis

OTUs, or taxonomic operating units, are the same markers artificially assigned to a taxon (strain, species, genus, group, etc.) for convenience of analysis in phylogenetic studies or population genetics studies. All sequences can be divided into OTUs according to different similarity levels, with each OTU corresponding to a representative sequence. We used Usearch software to cluster reads at a 97.0% similarity level to obtain our OTUs.

The total number of OTUs obtained in the test results was 475 (Figure 6). The numbers of OTUs in the CON group, L.S group, *E. coli* group and L.S + *E. coli* group were 453, 467, 463 and 467, respectively, with no significant differences. The Venn diagram (Figure 7) shows that the numbers of unique OTUs in the CON group compared with the *E. coli* group were 9 and 19, respectively, while the numbers of unique OTUs in the *E. coli* group and the L.S + *E. coli* group were 6 and 10, respectively.

#### 3.5.2. Species Distribution Histogram

With SILVA as the reference database, the naive Bayesian classifier was used to carry out taxonomic annotation on the feature sequence, and the species classification information corresponding to each feature can be obtained. Then, the community composition of each sample at each level (phylum, class, order, family, genus, species) was counted, and the species abundance table at different taxonomic levels was generated using QIIME software.

As shown in Figure 8, the distribution of species at class level (Figure 8A), family level (Figure 8B) and genus level (Figure 8C) was made according to the distribution of the relative content of species in the sample. The figure shows the species with the top 10 abundances.

At the class level (Figure 8A), the abundance ratios of *Bacteroidia* and *Gammaproteobacteria* in the *E. coli* group were significantly higher than those in the CON group, while the L.S + *E. coli* group demonstrated a significant decreasing trend. At the family level (Figure 8B), the abundance ratio of *Lachnospiraceae* in the *E. coli* group was significantly lower than that in the CON group, and the abundance ratio of *Muribaculaceae* and *Prevotellaceae* compared with the CON group increased significantly, while the L.S + *E. coli* group demonstrated the opposite trend of the *E. coli* group. At the genus level (Figure 8C), the abundance ratios of uncultured_bacterium_f_*Muribaculaceae* and *Alloprevotella* in the *E. coli* group were significantly higher than those in the CON group, while the abundance ratios of uncultured bacterium f *Lachnospiraceae* and *Ruminiclostridium* exhibited a downward trend, especially in the *Lachnospiraceae* NK4A136 group. Significantly, the L.S + *E. coli* group still demonstrated the opposite trend of the *E. coli* group.

#### 3.5.3. Alpha Diversity Analysis

The Chao1 and Ace indices measure species abundance and the number of species. The Shannon and Simpson indices are used to measure species diversity and are influenced by species abundance and species evenness in the sample community.

As shown in Figure 9A,B, compared with the *E. coli* group, the Ace index and the Chao1 index were higher in the L.S + *E. coli* group (*p* < 0.01, *p* < 0.05). As shown in Figure 9C,D, compared with the L.S group, the Shannon index and Simpson index were lower in the L.S + *E. coli* group (*p* < 0.05). It can be seen that ETEC K88 infection reduced the species abundance and species diversity of gut microbiota, and pretreatment with *Lactobacillus salivarius* WZ1 revised this situation.

#### 3.5.4. Beta Diversity Analysis

PCA and PCoA analyses were performed according to beta diversity based on distance. As shown in Figure 10A, the contribution rates of the two principal components to the sample differences were 27.56% and 13.28%, and there were differences among the groups, but the differences were not significant. As shown in Figure 10B, the contribution rates of the two principal components to the sample differences were 17.75% and 9.60%, respectively, and there were differences among the groups. There were differences between the *E. coli* group and the CON group, indicating that ETEC K88 changed the composition of the gut microbiota. In addition, there were also differences between the L.S + *E. coli* group and the *E. coli* group, indicating that *Lactobacillus salivarius* WZ1 modified the intestinal microbial population changed by ETEC K88.

#### 3.5.5. LEfSe Analysis

As shown in Figure 11, *Ralstonia* was the dominant species (LDA > 3) in the *E. coli* group, and its abundance was higher than that in the CON group. However, *Ralstonia* (LDA < 0) was not the dominant species in the L.S + *E. coli* group. In contrast, beneficial bacteria such as *Lactobacillus* (LDA > 2) and *Bifidobacterium* (LDA > 3) were the dominant species in the L.S + *E. coli* group. Therefore, ETEC K88 caused a negative change in the gut microbiota, while the addition of *Lactobacillus salivarius* WZ1 caused a positive change in the gut microbiota.

As shown in Figure 12A, ETEC K88 significantly decreased the abundance of *Lachnospiraceae*, and as shown in Figure 12B, ETEC K88 significantly increased the abundance of *Ralstonia*. These results indicate that ETEC K88 reduced the abundance of beneficial bacteria and increased the abundance of harmful bacteria in the gut.

#### 3.5.6. ANOVA Analysis

As shown in Figure 13, compared with the *E. coli* group, the relative abundances of *Ralstonia* and *Helicobacter* were lower in the L.S + *E. coli* group (*p* < 0.01), indicating that the diarrhea caused by ETEC K88 may be related to these two species, and *Lactobacillus salivarius* WZ1 effectively decreased these two harmful bacteria that cause diarrhea.

## 4. Discussion

The small intestine is an important part of the digestion and absorption of nutrients. The villi increase the contact area between the intestine and the nutrients, which is conducive to the absorption of more nutrients, and prevent colonization by harmful bacteria through their swaying motion [27,28]. Villus length, crypt depth and villus length to crypt depth (V/C) ratio are all important indicators of gut maturity and functional capacity. Therefore, the ratio of V/C corresponds to a relatively healthy gut system. We included high brush border enzyme activity, shorter villus length and lower intestinal absorptivity [29,30,31]. Compared with this experiment, ETEC K88 infection shortened the intestinal villi (*p* < 0.01) and increased the depth of the crypts (*p* < 0.01), resulting in a decrease in V/C (*p* < 0.01), suggesting that the absorption capacity of the small intestine was reduced, which is the key to causing diarrhea.

Experiments have shown that TLR4/NF-κB/MyD88 signaling is involved in the body’s inflammatory response [32,33], the transient overactivation of NF-κB–mediated signaling leads to acute inflammation, and the release of a series of pro-inflammatory cytokines, including *TNF-α*, *IL-1β* and *IL-6*, can lead to tissue damage [34]. This was consistent with the test results in the *E. coli* group, where the protein expressions of TLR4, NF-κB and MyD88 were increased (*p* < 0.01), and the mRNA expressions of *TNF-α*, *IL-1β* and *IL-6* were increased (*p* < 0.01). *Lactobacillus salivarius* can inhibit the adhesion of ETEC K88 to IPEC-J2 cells and, at the same time, has the ability to reduce the pro-inflammatory cytokines *IL-1β, TNF-α, IL-8* and TLR4 and significantly reduce the phosphorylation of p38 MAPK and p65 NF-κB, which indicates that *Lactobacillus salivarius* may reduce inflammation-related cytokines by inhibiting the phosphorylation of p38 MAPK and blocking the NF-κB signaling pathway [35]. *IL-6* has been reported to play a key role in the amplification of inflammatory signals in the gut, and it can inhibit the NF-κB pathway and pro-inflammatory cytokine *IL-6* production to reduce intestinal inflammation [36]. This is consistent with the test results showing that the protein expression of TLR4, NF-κB and MyD88 and the mRNA expression of *TNF-α*, *IL-1β* and *IL-6* in the L.S + *E. coli* group were lower than those of the *E. coli* group (*p* < 0.01). Studies have shown that TNF-α and IL-1β levels are also associated with the TLR4/NF-κB/MyD88 pathway [37]. This indicates that *Lactobacillus salivarius* WZ1 can inhibit the mRNA expression of *TNF-α, IL-1β* and *IL-6* in intestinal cells (*p* < 0.01) and the protein expression of the TLR4/NF-κB/MyD88 pathway (*p* < 0.01). Thus, the release of pro-inflammatory factors *TNF-α*, *IL-1β* and *IL-6* was reduced, and the intestinal inflammatory damage caused by ETEC K88 was finally inhibited.

The intestinal mucosa is composed of epithelial cells, which form an active barrier through the expression of pro-inflammatory genes, the secretion of inflammatory cytokines, and the recruitment of inflammatory cells to protect the subepithelial tissue from invasion by pathogenic bacteria, intestinal barrier dysfunction or intestinal passage. Increased permeability is an important condition in the pathogenesis of different diseases, and the main determinant of permeability is the integrity of intestinal epithelial tight junctions (TJs) [38,39]. An impaired intestinal tight junction (TJ) barrier is considered an important pathogenic factor in intestinal and systemic inflammation, and impairment of the intestinal tight junction (TJ) barrier can lead to paracellular infiltration by harmful pathogenic bacteria, thereby causing inflammatory responses, including Crohn’s disease, necrotizing enterocolitis, ulcerative colitis, alcoholic hepatitis and various infectious diarrhea syndromes [40]. Studies have shown that dietary supplementation of 0.1% and 0.2% *Lactobacillus salivarius* can significantly increase the levels of anti-inflammatory cytokines in serum and tight junction proteins Occludin, Claudin-1 and ZO-1 in LPS-stressed piglets, while serum pro-inflammatory factors TNF-α, IL-1β and IL-6 were significantly downregulated [41]. Multiple studies have shown that drug- or probiotic-induced enhancement of the intestinal epithelial TJ barrier prevents the development of intestinal inflammation in various mouse models of IBD [42,43,44,45]. This is consistent with the results of this experiment, where the protein expression levels of intestinal epithelial tight junction–related proteins Occludin, Claudin-1 and ZO-1 in the L.S + *E. coli* group were higher than those in the *E. coli* group (*p* < 0.01), and the mRNA expressions of TNF-α, IL-1β and IL-6 in the tissue were lower than those in the *E. coli* group (*p* < 0.01). Experiments have shown that the immune system plays an important role in regulating the function of the intestinal TJ barrier, and the opening of this barrier induced by pro-inflammatory cytokines is an important mechanism leading to TJ barrier damage under various inflammatory conditions in the intestine [46]. IL-1β, a typical pro-inflammatory cytokine, increases intestinal epithelial TJ permeability in animal and in vitro cell culture model systems [47]. Therefore, IL-1β plays an important role in intestinal inflammation, and its induced increase in intestinal epithelial TJ permeability is mediated by the p38 kinase activation of the ATF-2 and ATF-3 regulation of MLCK gene activity [48]. Furthermore, the IL-1β–induced increase in Caco-2 TJ permeability was mediated by NF-κB activation, and siRNA inhibition of NF-κB activation or NF-κB p65 depletion prevented IL-1β from increased Caco-2 TJ permeability. IL-1β also caused the downregulation of Occludin protein expression, decreased Occludin mRNA expression and interfered with Occludin junction localization, so the regulation of Occludin by IL-1β was also regulated by NF-κB activation [49]. This is consistent with the results of this experiment, where the expression of the NF-κB protein and the mRNA expression of IL-1β in the *E. coli* group were increased (*p* < 0.01), while the protein expression of Occludin was significantly decreased (*p* < 0.01).

The normal gut microbiota consists of 100 trillion different microbes, most of which are bacteria, including more than 1100 common species, with at least 160 in each individual [50]. Gut microbiota composition balance is important to ensure the stability of gut homeostasis, and microbiota dysbiosis is thought to play an important role in the pathogenesis of inflammatory bowel disease [51,52]. Trials have demonstrated bacterial dysbiosis in fecal samples from calves with diarrhea, with less diversity and fewer observable species in the diseased calves compared with the healthy controls [53]. It has also been reported that *Mycoplasma gallisepticum* complicated with *Escherichia coli* infection significantly reduces the Chao1 index of intestinal microbes, while an intake of *Lactobacillus salivarius* improves the composition of intestinal microbes [54]. Combined with the alpha diversity analysis results of this experiment, the Ace index of the *E. coli* group was lower than that of the L.S + *E. coli* group (*p* < 0.01), and the Chao1 index was lower in the L.S + *E. coli* group (*p* < 0.05). This indicates that ETEC K88 infection may reduce the population of beneficial bacteria and reduce the species abundance and species diversity of gut microbiota, while the addition of *Lactobacillus salivarius* WZ1 improves this situation. Experiments have shown that *Escherichia coli*, *Enterobacter* and *Enterococcus* rapidly proliferate in feces after 7 days of *Escherichia coli* infection in mice [55]. This is consistent with the experimental results showing that the abundance ratios of *Bacterodia* and *Gammaproteobacteria* in the *E. coli* group were significantly higher than those in the CON group. The oral administration of *Lactobacillus salivarius* to suckling pigs for 10 days can increase the number of fecal lactobacilli [56]. This is consistent with the results showing that the addition of *Lactobacillus salivarius* WZ1 in the LEfSe analysis can increase the abundance of *Lactobacillus* in the gut, indicating that *Lactobacillus salivarius* WZ1 can increase the abundance of beneficial bacteria in the gut, thereby inhibiting the growth of harmful bacteria and protecting gut health. In conclusion, *Lactobacillus salivarius* WZ1 can inhibit the inflammatory damage caused by ETEC K88 in the mouse jejunum by regulating the TLR4/NF-κB/MyD88 inflammatory pathway and gut microbiota.

## 5. Conclusions

*Lactobacillus salivarius* WZ1 can inhibit the inflammatory damage caused by ETEC K88 in the mouse jejunum by regulating the TLR4/NF-κB/MyD88 inflammatory pathway. It can also promote the secretion of intestinal epithelial tight junction protein, enhance the intestinal epithelial barrier, and increase the abundance of beneficial bacteria in the intestinal tract, including *Lactobacillus* and *bifidobacterium*.

The bacteriostatic substances produced by *Lactobacillus salivarius* WZ1 will be further studied and purified. This will lay a foundation for clinical use of *Lactobacillus salivary* to prevent calf diarrhea in the future.

## Figures and Tables

**Table 1:** Primers for real-time PCR analyses.

Primer	Primer Sequences
*IL-1β* F	5′–TTCAGGCAGGCAGTATCACTC–3′
*IL-1β* R	5′–GAAGGTCCACGGGAAAGACAC–3′
*IL-4* F	5′–GGTCTCAACCCCCAGCTAGT–3′
*IL-4* R	5′–GCCGATGATCTCTCTCAAGTGAT–3′
*IL-6* F	5′–CTGCAAGAGACTTCCATCCAG–3′
*IL-6* R	5′–AGTGGTATAGACAGGTCTGTTGG–3′
*TNF-α* F	5′–CAGGCGGTGCCTATGTCTC–3′
*TNF-α* R	5′–CGATCACCCCGAAGTTCAGTAG–3′
*GAPDH* F	5′–GGGAAGCCCATCACCATCTT–3′
*GAPDH* R	5′–GCCTTCTCCATGGTGGTGAA–3′

## Data Availability

Data are contained within the article.
